# Humanin analogue, HNG, inhibits platelet activation and thrombus formation by stabilizing platelet microtubules

**DOI:** 10.1111/jcmm.15151

**Published:** 2020-03-16

**Authors:** Lijie Ren, Qing Li, Tao You, Xuefei Zhao, Xingshun Xu, Chaojun Tang, Li Zhu

**Affiliations:** ^1^ Suzhou Key Laboratory of Thrombosis and Vascular Diseases State Key Laboratory of Radiation Medicine and Protection Cyrus Tang Hematology Center Collaborative Innovation Center of Hematology of Jiangsu Province Soochow University Suzhou China; ^2^ The Institute of Neuroscience Soochow University Suzhou China

**Keywords:** humanin, microtubule, platelet activation, platelet aggregation, thrombus formation

## Abstract

HNG, a highly potent mutant of the anti‐Alzheimer peptide‐humanin, has been shown to protect against ischaemia‐reperfusion (I/R) injury. However, the underlying mechanism related to platelet activation remains unknown. We proposed that HNG has an effect on platelet function and thrombus formation. In this study, platelet aggregation, granule secretion, clot retraction, integrin activation and adhesion under flow conditions were evaluated. In mice receiving HNG or saline, cremaster arterial thrombus formation induced by laser injury, tail bleeding time and blood loss were recorded. Platelet microtubule depolymerization was evaluated using immunofluorescence staining. Results showed that HNG inhibited platelet aggregation, P‐selectin expression, ATP release, and α_IIb_β_3_ activation and adhesion under flow conditions. Mice receiving HNG had attenuated cremaster arterial thrombus formation, although the bleeding time was not prolonged. Moreover, HNG significantly inhibited microtubule depolymerization, enhanced tubulin acetylation in platelets stimulated by fibrinogen or microtubule depolymerization reagent, nocodazole, and inhibited AKT and ERK phosphorylation downstream of HDAC6 by collagen stimulation. Therefore, our results identified a novel role of HNG in platelet function and thrombus formation potentially through stabilizing platelet microtubules via tubulin acetylation. These findings suggest a potential benefit of HNG in the management of cardiovascular diseases.

## 1 INTRODUCTION

Humanin (HN), a 24‐amino acid bioactive peptide isolated from the brains of patients with Alzheimer's disease (AZD),^1^ has been shown to protect neurons from cell death.^2^, ^3^ A bulk of the evidence suggests that HNG, a 1000‐fold more potent analogue of HN, significantly ameliorates amyloid beta (Aβ)‐induced cytotoxicity and neurological dysfunction.^4^, ^5^, ^6^, ^7^, ^8^, ^9^ HNG was found to inhibit the fibrogenesis of Aβ in vitro and to attenuate tissue amyloid deposition in animals after long‐term administration.^5^, ^10^ Interestingly, a recent report revealed that acute HNG treatment also decreases brain Aβ accumulation and tau protein hyperphosphorylation induced by cardiac I/R in rats.^11^

Platelets play an essential role in physiological haemostasis and pathological thrombosis and are also linked to neurodegenerative diseases, such as AZD.^12^ Resting platelets in circulation maintain their discoid shape. Upon stimulation, platelets are activated followed by secreting intracellular granules that contain pro‐inflammatory and pro‐coagulant factors including Aβ40. The deposition of Aβ peptides, mainly Aβ40 in the vessel wall, causes the vascular destruction and contributes to the severity of AZD pathology.^13^ Activated platelets are the main source of circulating Aβ40.^14^, ^15^ Aβ40 in turn activated platelets and enhanced platelet aggregation via integrin αIIbβ3 and the intracellular signalling pathway, further deteriorated cerebral diseases.^13^

The protection role of HNG led us to propose that platelets might be a potential target of HNG. To date, whether and how HNG affects platelet function has not yet been reported. Moreover, the role of anti‐AZD agents in thrombotic disorders is far less understood. Given the emerging signalling nexus between AD and thrombosis, it is tempting to assume a common therapeutic target. Therefore, we have been suggested that HNG, a neuroprotective peptide, may inhibit platelet activity. In the current study, we demonstrated that HNG inhibits platelet activation and thrombus formation by stabilizing microtubules and enhancing tubulin acetylation.

## 2 METHODS AND MATERIALS

### 2.1 Animals

Wild‐type (WT) C57BL/6J mice were housed in a specific pathogen‐free environment with constant day/night light cycles and were given free access to food and water. Mice aged 8 to 10 weeks were used for all experiments. The experimental protocols were approved by the Institutional Animal Care and Use Committee of Soochow University and conformed to the National Institutes of Health Guide for the Care and Use of Laboratory Animals (National Institutes of Health, 8th edition, 2011).^16^

### 2.2 Antibodies and reagents

Peptide HNG was synthesized from Shanghai Bootech Bioscience and Technology Co., Ltd. Anti‐acetylated tubulin antibody (T6793) and anti‐β1 tubulin antibody (ab179511) were purchased from Sigma‐Aldrich and Abcam. Anti‐phospho‐PLCγ2 (Y1217), anti‐phospho‐ERK1/2 (Thr202/Tyr204), anti‐phospho‐p38 MAPK (Thr180/Tyr182), anti‐phospho‐Akt (Ser473), anti‐PLCγ2, anti‐ERK1/2, anti‐p38 MAPK and anti‐Akt antibodies were purchased from Cell Signaling Technologies. Phalloidin‐TRITC was obtained from Sigma‐Aldrich. PE‐conjugated mouse anti‐human CD62P antibody and FITC‐conjugated PAC‐1 antibody were purchased from BD Biosciences.

### 2.3 Isolation of human platelets

Venous blood was drawn from healthy donors, anticoagulated with 1/5 volume ACD buffer and centrifuged at 900 r.p.m. for 20 minutes to yield PRP, which was applied to a column packed with Sepharose^TM^ 2B resin. Platelets were eluted, counted, pooled and adjusted to a concentration of 2‐3 × 10^8^/mL with Tyrode's buffer and allowed to equilibrate at RT for 30 minutes before analyses. All human studies were in accordance with the Declaration of Helsinki and approved by the ethics committee of Soochow University.^17^

### 2.4 Platelet aggregation and ATP release

Gel‐filtered platelets were incubated with HNG or vehicle solution (ddH_2_O) in glass cuvettes for 10 minutes at 37°C and stimulated with collagen (2 μg/mL), thrombin (0.01 U), convulxin 0.2 nM, ADP (10 μM) or CRP (0.2 μg/mL) in the presence of CaCl_2_ (1 mM) under stirring conditions (1000 r.p.m.). Platelet aggregation and ATP release (nmols) were monitored using an aggregometer (Chrono‐Log). ATP standard (final concentration: 2 nM) was used to normalize the released ATP.

### 2.5 Activated integrin α_IIb_β_3_, P‐selectin expression and clot retraction

Gel‐filtered human platelets were diluted to 5 × 10^7^/mL using Tyrode's buffer; incubated with HNG (10 µM), scramble‐HNG (10 µM) or vehicle for 10 minutes at 37°C; and stimulated with CRP (1 or 2 μg/mL) or thrombin (0.05U) in the presence of FITC‐labelled soluble fibrinogen, FITC‐labelled PAC‐1 or PE‐labelled CD62P antibody at RT. After 15 minutes, platelets were fixed with 2% paraformaldehyde, and surface FITC‐ or PE‐fluorescence was analysed using flow cytometry.

For clot retraction, human platelets were stimulated with thrombin (1U) and recorded at an indicated time‐point using a camera. The clot area was quantified by the ratio of clot area to platelet suspension area at different time‐points.

### 2.6 Adhesion of platelets under flow conditions

Platelet adhesion under shear was measured using a BioFlux200^TM^ flow chamber system (Fluxion Biosciences Inc) according to the manufacturer's instructions. Channels in BioFlux^TM^ plates were primed, coated with type I collagen (200 µg/mL, Chrono‐log) for 1 h at RT and blocked with 0.5% BSA/PBS. Sodium citrate‐anticoagulated human whole blood was pre‐incubated with HNG (10 μM), labelled with calcein‐AM (10 μM, Molecular Probes) for 30 minutes and perfused through the channels at 10 dyne/cm^2^. Adherent platelets were quantified by fluorescence recorded under a ZEISS inverted microscope equipped with a monochrome digital camera.

### 2.7 Platelet spreading and microtubule depolymerization

Glass coverslips were precoated with fibrinogen. After incubation with HNG or vehicle at 37°C for 10 minutes, platelets were allowed to spread on coated coverslips in 48‐well plates for 60 minutes. After washing, adherent platelets were fixed with 4% paraformaldehyde, permeabilized with 0.1% Triton X‐100 and stained with TRITC‐labelled phalloidin at RT for 2 h. Coverslips were mounted on glass slides, and pictures were obtained under a fluorescence microscope (Olympus, FSX100) and were analysed using ImageJ software (NIH).

For the platelet microtubule acetylation assay, resting platelets or microtubule depolymerization reagent, nocodazole‐treated platelets after incubation with HNG (10 μM) at 37°C for 10 minutes were fixed in microtubule‐preserving fixative (PHEM) buffer supplemented with 4% PFA (1:1), followed by centrifugation on poly‐L‐lysine‐coated coverslips. Alternatively, platelets were incubated with HNG and then allowed to adhere to fibrinogen‐coated slides for 1 h before fixation. Samples were stained using an anti‐β1‐tubulin (T4026, clone TUB 2.1; Sigma‐Aldrich) or anti‐acetylated tubulin antibody (T6793; Sigma‐Aldrich) and examined using a Leica TCS SP5 confocal microscope (Leica Microsystems). Assays were performed in biological triplicates. The number of platelets with intact microtubule rings was analysed using ImageJ.

### 2.8 Tail bleeding, blood loss and jugular vein bleeding

Tail bleeding times were determined as previously described.^18^ Briefly, HNG (final concentration in whole blood was about 10μM) or saline was administered through the jugular vein to mice. Ten minutes later, the mice were placed on a heating pad, and their tails protruded. After transection of the distal 3 millimetres, the tail was immediately immersed in 13 ml of 0.9% sodium chloride for 30 minutes at 37°C. The time to the first bleeding cessation was recorded. After 30 minutes, each tube is then centrifuged at 550 g during 5 minutes. After the removal of the supernatant, a lysis buffer (NH4Cl 150 mM, KHCO3 1 mM, EDTA 0.1 mM, pH 7.2) was added on the clot for a final volume of 2 ml. The tubes were vortexed, and the absorbance at 540nm was measured.

For the jugular vein bleeding time test, the other jugular vein was stabbed using a needle and then washed with 0.9% sodium chloride. The time to bleeding cessation was recorded.

### 2.9 Laser‐induced cremaster arterial thrombus formation by intravital microscopy

Wild‐type male mice aged 8‐12 weeks were anaesthetized with 1% sodium pentobarbital via intraperitoneal (i.p.) injection. A cannula was inserted into the jugular vein to infuse HNG (10 μM) or saline and 3,3’‐dihexyloxacarbocyanine iodide (DIOC6, 200 μM) before laser injury. Ten minutes later, arterioles (30‐40 µm) were injured using a Laser Ablating system (Intelligent Imaging Innovation). Digital images were captured by a Zeiss camera for 4 minutes after vessel wall injury. Data from 26 thrombi per group were analysed using Slidebook version 6.0 software.

### 2.10 Western blotting

Pre‐incubated platelets (250 µl, 2 × 10^8^/ml) were stimulated by agonist with stirring. At the indicated time‐points, the reaction was terminated by adding radioimmunoprecipitation assay (RIPA) buffer supplemented with protease and phosphatase inhibitor cocktails and lysed for 30 minutes on ice. After centrifugation, supernatants were collected, and protein concentrations were determined using the BCA method. Samples were separated on a 10% SDS‐PAGE gel and incubated with the indicated primary antibody and corresponding fluorescence‐conjugated secondary antibodies (goat anti‐rabbit IRDye 800CW, goat antimouse IRDye 800CW; CST). Fluorescence intensity was recorded using an Odyssey infrared imaging system (LI‐COR Biosciences). Densitometry analysis was performed using ImageJ software.

### 2.11 Statistics

Data are expressed as the means ± standard errors of the mean (SEMs) or medians with interquartile ranges (IQRs). Comparison of the differences among multiple groups was performed using one‐way analysis of variance (ANOVA) with the Dunnett's multiple comparisons test. A two‐tailed Student's *t* test or Mann‐Whitney test was used compare the differences in variables with or without a normal distribution. Statistical analysis was performed using Prism 8.0 software (GraphPad Software). *P* values <.05 were considered statistically significant.

## 3 RESULTS

### 3.1 HNG inhibits platelet aggregation and granule release

**Figure 1:**
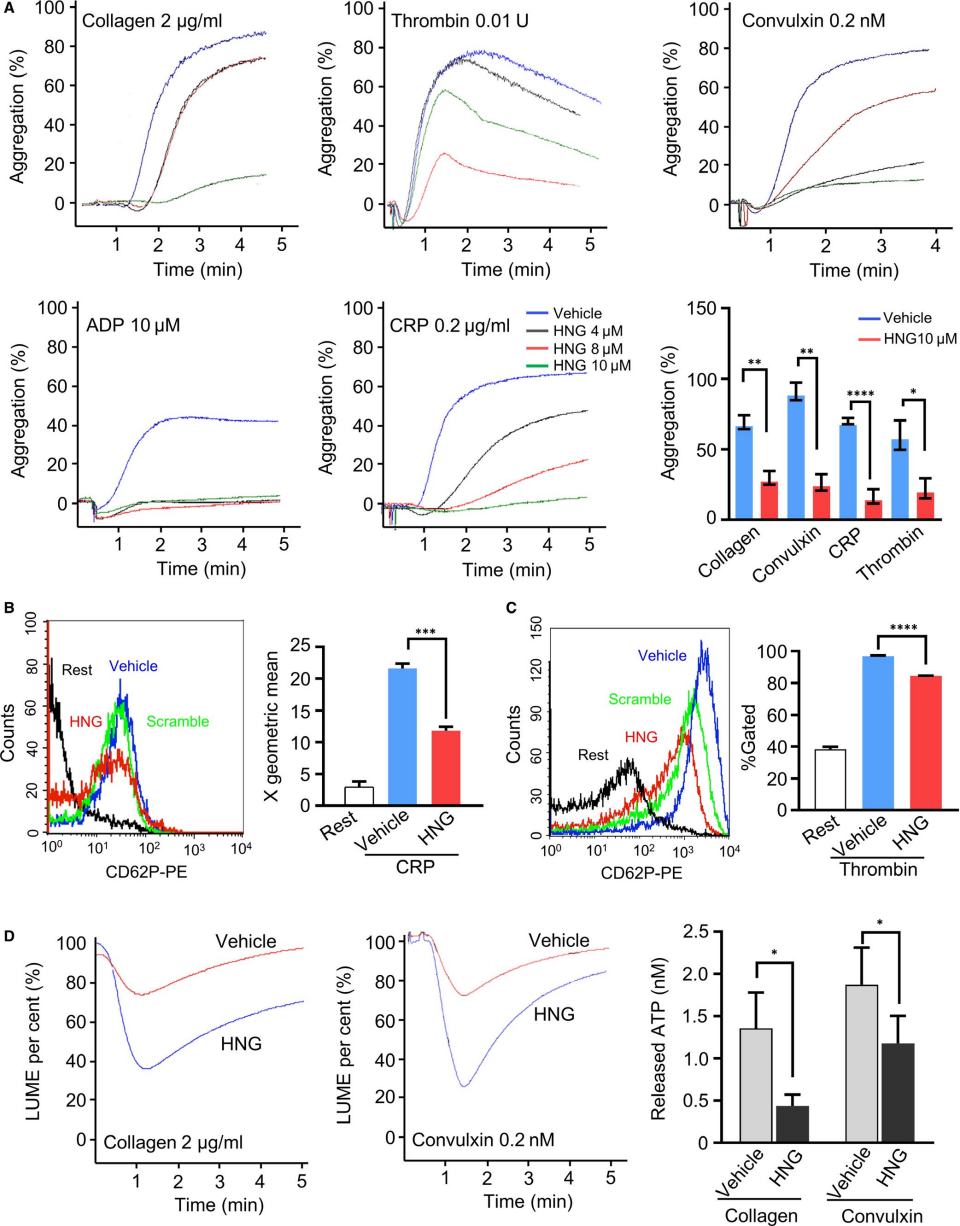
HNG impairs platelet aggregation and granule release. (A) Gel‐filtered human platelets were pre‐incubated with HNG (4 μM, 8μM or 10 μM) or vehicle solution (ddH_2_O) for 10 minutes and stimulated with collagen 2 µg/mL, thrombin 0.01 U/mL, convulxin 0.2 nM, ADP 10 μM and CRP 0.2 µg/mL, respectively. Statistical data of HNG10μM and vehicle were shown. *P < .05, **P < .01, ***P < .001, ****P < .0001, N > 3, unpaired t test. (B, C) The surface expression of P‐selectin was analysed by flow cytometry. 10 μM HNG or scramble‐HNG was pre‐incubated with gel‐filtered human platelets for 10 minutes at 37℃ and then stimulated by 2 μg/mL CRP (B) or 0.05U thrombin (C). A representative fluorescent histogram of PE‐conjugated P‐selectin is shown. Statistical data were analysed using X geometric mean fluorescence and the percentage of gated cells. ***P < .001, ****P < .0001, ordinary one‐way ANOVA, Dunnett's multiple comparisons test. (D) ATP released from platelets was monitored using an aggregometer. After incubation with HNG or vehicle, gel‐filtered platelets were induced by 2 µg/mL collagen or 0.2 nM convulxin. ATP standard (final concentration: 2 nM) was used to normalize the released ATP. *P < .05, mean ± SEM, N > 3, paired t test

To test the effect of HNG on platelet function, isolated human platelets were pre‐incubated with HNG or vehicle and stimulated with various agonists. The results showed that HNG inhibited platelet aggregation simulated by collagen, convulxin, collagen‐related peptide (CRP), thrombin or ADP (Figure 1A). Quantification of the maximal inhibition percentage of platelet aggregation at 10μM HNG treatment showed that the inhibitions are significant compared to the vehicle (collagen, *P* < .01; convulxin, *P* < .01; CRP, *P* < .001; thrombin, *P* < .05). As collagen, convulxin and CRP shared GPVI signalling pathway and caused similar inhibition on platelet aggregation (Figure 1A), we use collagen or CRP in the following experiments. As HNG inhibited ADP‐ and thrombin‐induced platelet aggregation, thrombin was used for the following experiments as well.

Activated platelets release α‐granule containing P‐selectin, a PSGL‐1 ligand, mediating platelet‐leucocyte or leucocyte‐endothelial interaction.^19^, ^20^ To determine whether HNG affects this process, human platelets pre‐incubated with 10μM HNG were stimulated by platelet agonists CRP and thrombin, and cell surface P‐selectin expression was measured using flow cytometry (Figure 1B,C). Compared with vehicle, HNG significantly inhibited the increment of P‐selectin in CRP (Figure 1B, *P* < .001) and thrombin (Figure 1C, *P* < .0001) treated platelets. Next, we measured the secretion of ATP, a strong pro‐inflammatory factor and a hallmark of platelet dense granule release. Similarly, HNG inhibited ATP release from collagen‐ and convulxin‐stimulated platelets (Figure 1D, *P* < .05). Moreover, we also showed that HNG did not affect platelet viability and have no peptide cytotoxicity at high doses (20 μM) (Figure S1). Therefore, HNG showed an inhibitory effect on platelet aggregation and granule secretion from activated platelets without affecting platelet viability.

### 3.2 HNG inhibits platelet integrin α_IIb_β_3_ activation, spreading and clot retraction

**Figure 2:**
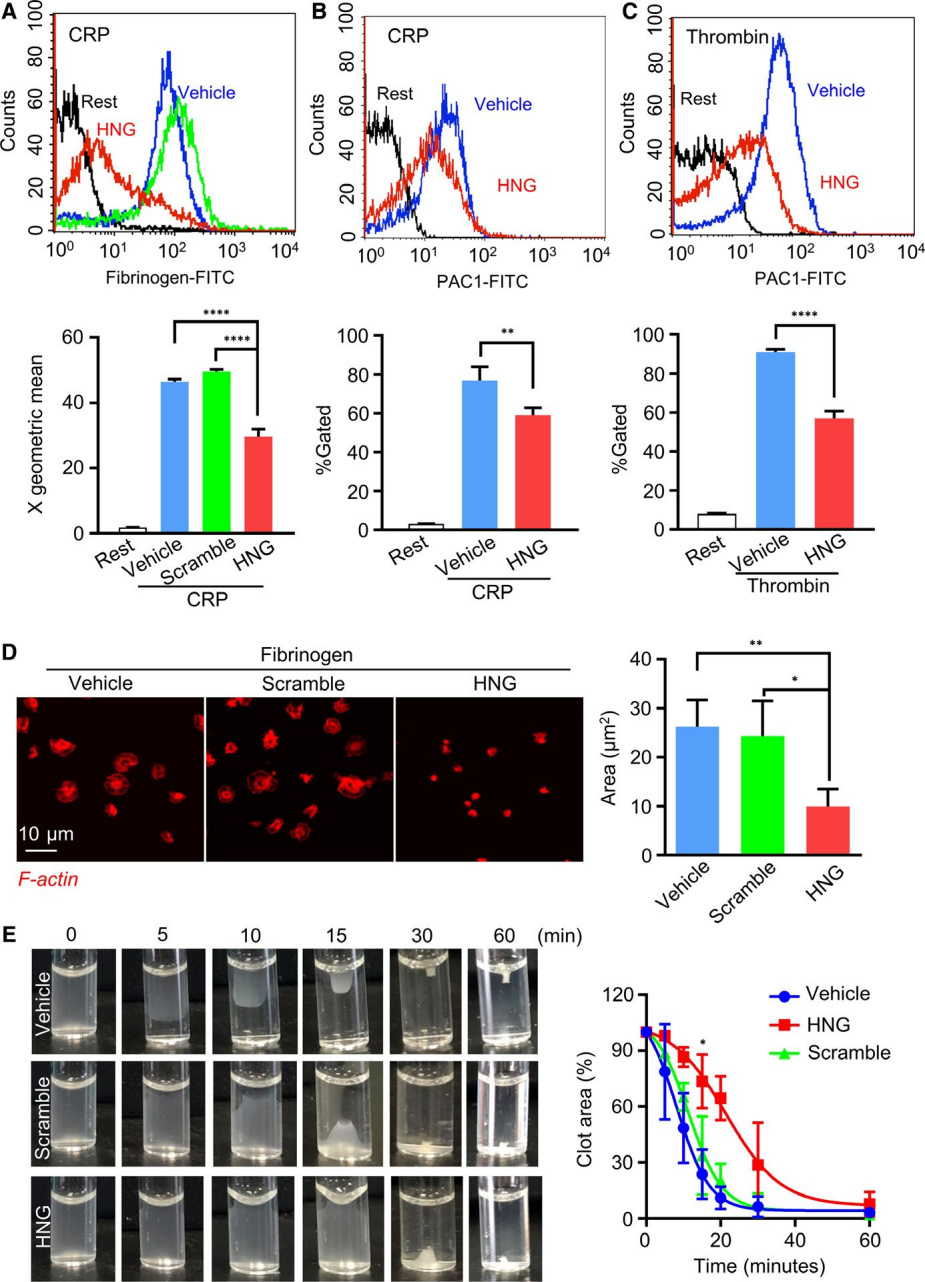
HNG inhibits platelet integrin αIIbβ3 activation, spreading and clot retraction. (A‐C) Human platelets were pre‐incubated with 10μM HNG, scramble‐HNG (10μM) or vehicle for 10 minutes at 37℃, and the fluorescence of FITC‐conjugated fibrinogen binding (A) or FITC‐conjugated PAC‐1 (B, C) was measured by flow cytometry after stimulated by 1μg/ml CRP or 0.05U thrombin. A representative histogram is shown. Statistical data were analysed using X geometric mean fluorescence or the percentage of gated cells. **P < .01, ****P < .0001, ordinary one‐way ANOVA, Dunnett's multiple comparisons test. (D) Effect of HNG on platelet spreading. 10µM HNG or scramble‐HNG‐treated platelets were placed on fibrinogen‐coated glass coverslips for 1 h at 37℃. After washing with PBS to remove non‐adherent platelets, adhered platelets were stained with TRITC‐labelled phalloidin. Images were obtained with an Olympus fluorescence microscope. Representative images are shown. Statistical data were calculated from the mean of the average surface area of individual platelets. *P < .05, **P < .01, N > 3, ordinary one‐way ANOVA, Dunnett's multiple comparisons test. (D) HNG impairs platelet clot retraction. After incubation with 10 µM HNG or vehicle, human platelets were stimulated with 20 µg/mL fibrinogen and 1 U thrombin and recorded at the indicated time‐point using a camera. The clot area was quantified by the ratio of clot area to platelet suspension area at different time‐points. *P < .05, unpaired t test

Given its inhibitory effect on platelet aggregation and secretion, we then asked whether HNG affects the ‘inside‐out’ conformation change of integrin α_IIb_β_3_, a pivotal glycoprotein required for the later phase of platelet activation and cytoskeletal modification.^21^ After preincubation with 10μM HNG or vehicle, human platelets were incubated with FITC‐conjugated soluble fibrinogen or FITC‐conjugated PAC‐1 antibody, which recognizes an epitope on α_IIb_β_3_ of activated platelets at or near the platelet fibrinogen receptor before stimulation. Then, activated integrin was measured using flow cytometry. The results demonstrated that HNG significantly reduced fibrinogen binding induced by CRP (Figure 2A, *P* < .0001), and the activated form of α_IIb_β_3_ (FITC‐conjugated PAC‐1) had a significant shift stimulated by CRP (Figure 2B, *P* < .01) and thrombin (Figure 2C, *P* < .0001) in human platelets. Taken together, these results indicate an inhibitory effect of HNG on integrin α_IIb_β_3_ activation.

Full platelet activation and stable thrombus formation require the reassembly of the cytoskeleton downstream of integrin ligation. To assess the role of HNG in this ‘outside‐in’ signal transduction, we evaluated platelet spreading on immobilized fibrinogen by labelling the actin skeleton with phalloidin. Compared with vehicle or scramble‐HNG, HNG reduced the average spreading area of adherent platelets (Figure 2D, *P* < .01). Integrin α_IIb_β_3_‐mediated ‘outside‐in’ signals lead to platelet clot retraction.^22^ To test the role of HNG in platelet clot retraction, human platelets were incubated with fibrinogen and thrombin. Compared with vehicle or scramble‐HNG, HNG delayed platelet clot retraction and significantly inhibited clot retraction 15 min after thrombin stimulation (Figure 2E, *P* < .05). Taken together, HNG inhibited integrin α_IIb_β_3_‐mediated ‘outside‐in’ signalling.

### 3.3 HNG inhibits thrombus formation without impairing haemostasis

**Figure 3:**
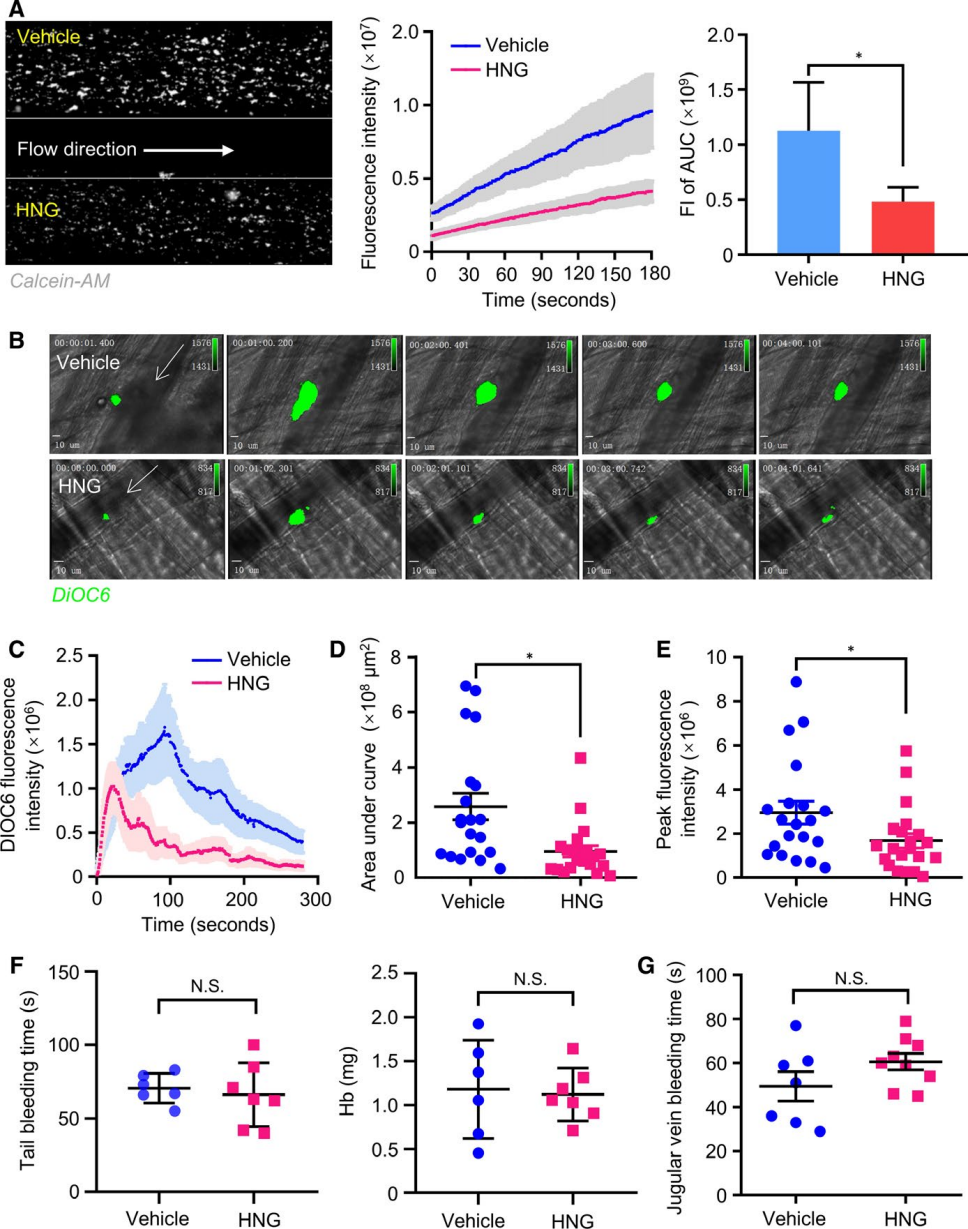
HNG inhibits thrombus formation without impairing haemostasis. (A) 10 µM HNG inhibits the flow‐associated platelet adhesion on collagen (100 µg/mL). HNG‐ or vehicle‐treated human whole blood was labelled with calcein‐AM (10 µM) for 30 min and perfused through the channels at 10 dyne/cm^2^. Live fluorescence was recorded. *P < .05, paired one‐tail t test, fluorescence of area under curve (AUC), vehicle versus HNG. (B‐E) HNG impaired laser‐induced mouse cremaster arterial thrombus formation. HNG (10 µM) or saline was infused into the jugular vein of male C57 mice using a cannula. The thrombus was visualized by 3,3′‐dihexyloxacarbocyanine iodide (DIOC6) staining and monitored real‐time under an intravital microscope (B). The median thrombus fluorescence intensity curve (C), the area under the curve (D) and the peak fluorescence intensity (E) were analysed. *P < .05, mean ± SEM, number of thrombus: 25‐26. (F‐G) HNG (10 μM) or vehicle was administered through the jugular vein to mice. The tail bleeding, blood loss (F) and jugular vein bleeding time (G) in mice were recorded. NS, *P* > .05, unpaired t test

We showed that HNG inhibited platelet function under static or stirring conditions. However, whether these effects persist under shear stress remains unclear. To study whether HNG inhibits flow‐associated adhesion, we labelled HNG‐treated human platelets with calcein‐AM and perfused them through collagen‐coated microfluidic channels. Live fluorescence monitoring showed that HNG significantly decreased the number of adherent platelets on the immobilized collagen matrix compared with vehicle (Figure 3A, fluorescence area under the curve, *P* < .05).

Next, we evaluated the role of HNG as a potential antithrombotic agent in a laser‐induced mouse cremaster arterial injury model with an intravital microscope. HNG or saline was infused into the jugular vein of male C57 mice using a cannula. The thrombus was visualized by 3,3′‐dihexyloxacarbocyanine iodide (DIOC6) staining and monitored real‐time under an intravital microscope (Figure 3B). The median thrombus fluorescence intensity curve (Figure 3C), the area under the curve (Figure 3D) and the peak fluorescence intensity (Figure 3E) showed that HNG attenuates thrombus formation in laser injury of the cremaster arterial (*P* < .05). Furthermore, we evaluated the impact of HNG on normal haemostasis. Tail bleeding time in mice was recorded. The results showed that HNG did not prolong the tail bleeding time and did not increase the blood loss compared with that of vehicle group (Figure 3F, *P* > .05). In addition, we also found that there were no significant changes in the mouse jugular vein bleeding time in the HNG group (Figure 3G, *P* > .05) compared with that in the control group, suggesting a minimal effect of HNG on blood coagulation.

### 3.4 HNG stabilizes platelet microtubules by enhancing tubulin acetylation and inhibits AKT and ERK phosphorylation downstream of HDAC6

**Figure 4:**
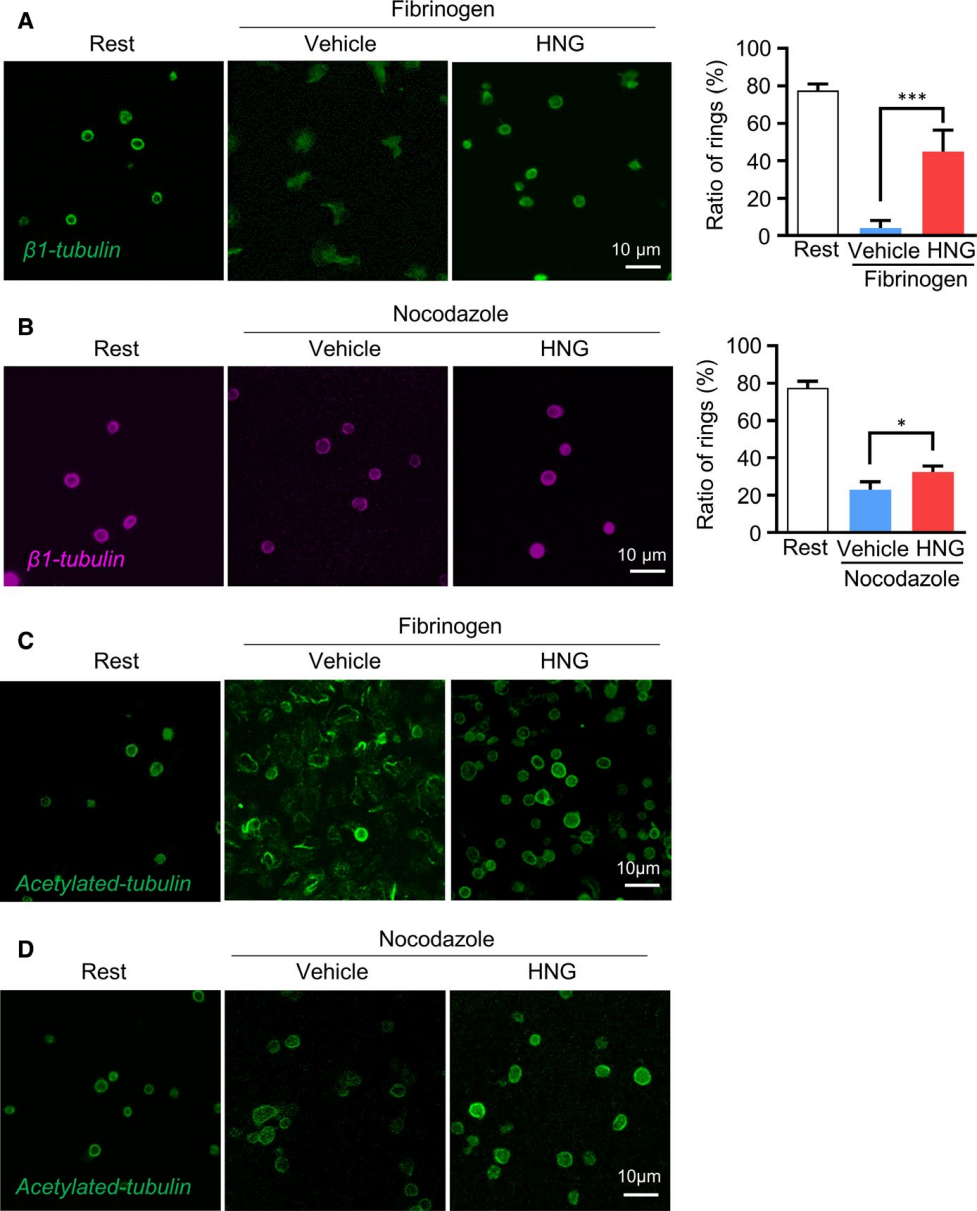
HNG stabilizes platelet microtubules from tubulin deacetylation. (A‐B) Resting platelets or microtubule depolymerization reagent, nocodazole‐treated platelets after incubation with 10 μM HNG, scramble‐HNG or vehicle at 37°C for 10 minutes were centrifugated on poly‐L‐lysine‐coated coverslips. Alternatively, platelets were incubated with HNG and then allowed to adhere to fibrinogen‐coated slides for 1 h at 37℃ before fixation. Samples were stained with β1‐tubulin (A, B) and acetylated tubulin (C, D) antibodies. Representative images are shown. Microtubule stability was analysed using the ratio of ring structure (A, B). *P < .05, ***P < .001, ordinary one‐way ANOVA, Dunnett's multiple comparisons test

Tubulin acetylation has been recognized as an important event in platelet activation via co‐ordinating cytoskeleton changes and signalling transduction. Inhibition of the deacetylase HDAC6 inhibits platelet aggregation and Rho‐kinase activation.^23^ To determine whether HNG stabilizes platelet microtubules by enhancing tubulin acetylation, we evaluated the number of microtubule coils (ring structure), designated as the marginal band, and tubulin acetylation in platelets. As shown in Figure 4A, resting platelets maintained intact marginal band. Activation of platelets led to the depolymerization of microtubules. After preincubation with HNG, a higher number of microtubule ring structure in fibrinogen‐stimulated were observed compared to the vehicle (Figure 4A, *P* < .001). Furthermore, nocodazole, a microtubule depolymerization reagent, was used to depolymerize microtubules without activating platelets. Results showed that HNG‐treated platelets preserved ring structure compared to the vehicle (Figure 4B, *P* < .05), suggesting that HNG increases platelet microtubule stability. When stained with anti‐acetylation antibody, preincubation with HNG preserved microtubule ring structure by maintaining tubulin acetylation in fibrinogen‐, nocodazole‐, CRP‐, or thrombin‐stimulated platelets (Figure 4C,D and Figure S2).

As previous studies have suggested that defective integration of tubulin subunits may alter microtubule stability, we further evaluated the potential impact of HNG on the incorporation of β1‐tubulin with α‐tubulin. As shown in Figure S3 most β1‐tubulin was colocalized with α‐tubulin in HNG‐treated platelets, and there was a persistent marginal ring. Taken together, these data suggest that HNG is a positive regulator of tubulin acetylation, although microtubule composition is unchanged.

**Figure 5:**
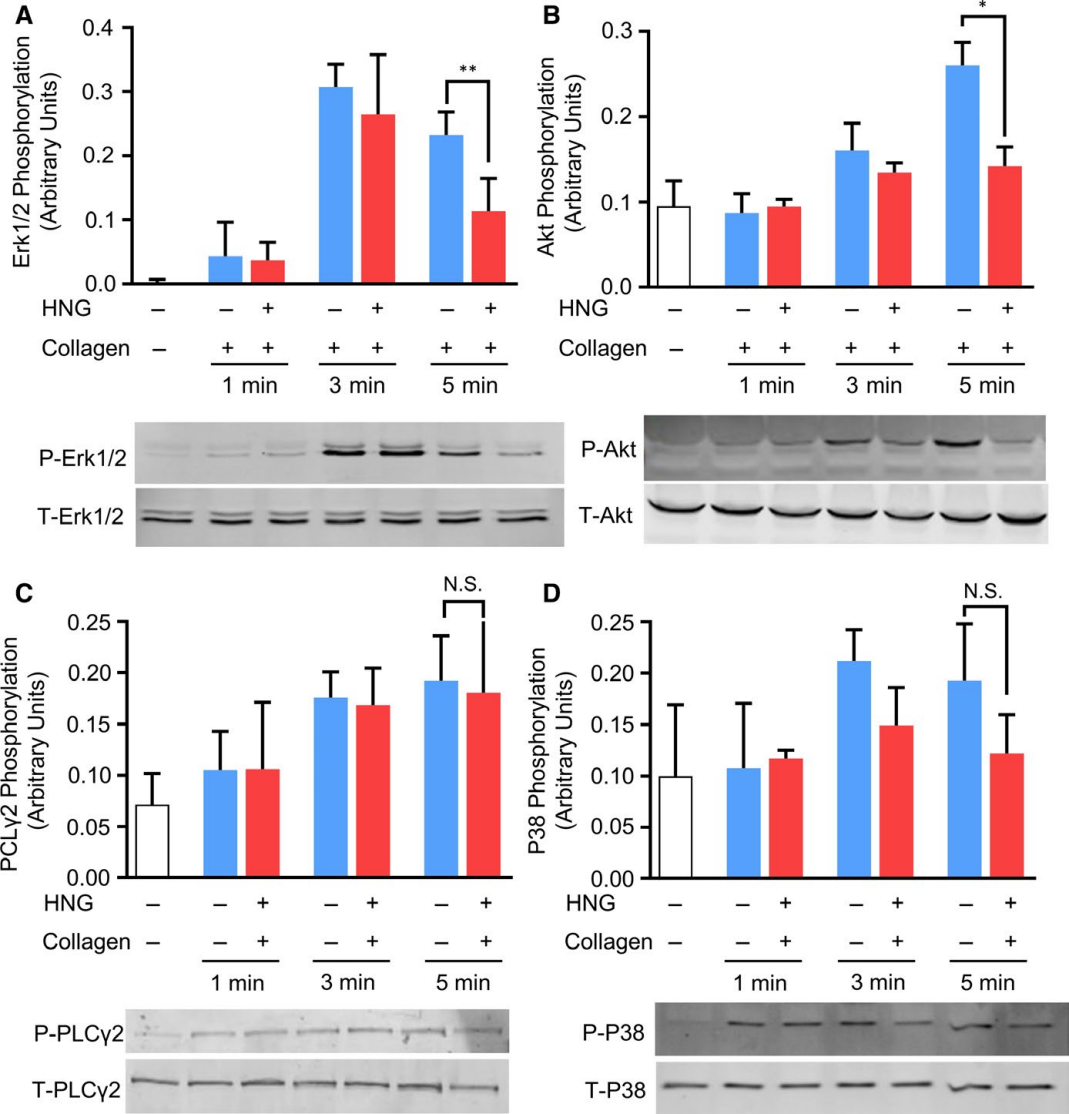
HNG inhibits AKT and ERK phosphorylation downstream of HDAC6. Gel‐filtered human platelets were pre‐incubated with 10 μM HNG for 10 minutes at 37℃ and then stimulated with 2 μg/mL collagen at different time‐points under stirring. After lysis with RIPA buffer, samples were analysed by Western blot, and ERK1/2 (A), AKT (B), PLCgamma2 (C) and P38 (D) phosphorylation was detected using antibodies and quantified. *P < .05, **P < .01, *t* test

HDAC6 is identified as the canonical enzyme responsible for deacetylating platelet tubulin.^24^ It acts as a signal mediator connecting early tyrosine kinase activation and downstream Rho GTPase signalling. Through the P21‐activated kinase PKA, Rho GTPase RAC activates ERK1/2 and AKT in platelets, giving rise to the reorganization of the platelet cytoskeleton.^23^ To determine that HNG may inhibit the activity of HDAC6, we measured the phosphorylation of ERK1/2 and AKT in collagen‐activated platelets. Consistent with the increased tubulin acetylation observed in Figure 4, platelets in the HNG group showed attenuated activation of ERK1/2 (Figure 5A, *P* < .01) and AKT (Figure 5B, *P* < .05) at 5 minutes after stimulation. On the other hand, agonist‐induced phosphorylation of PLC gamma 2 and P38MAPK was not changed by HNG (Figure 5C,D). These data implied that HNG‐mediated tubulin stabilization might be attributed to the inhibition of HDAC6.

## 4 DISCUSSION

HNG is a derivative of humanin with serine‐14 substitution by glycine, which potently enhances its neuroprotective activity.^1^ In this study, we demonstrated that HNG inhibits platelet activation and thrombus formation probably via stabilizing platelet microtubules. To the best of our knowledge, this is the first report on the link between HNG and platelet function.

For more than one decade, emerging evidence has suggested HNG as a multifunctional peptide with antiapoptotic, anti‐inflammatory, antioxidant and mitochondrial protective potencies.^5^, ^7^, ^25^, ^26^ Moreover, HNG showed substantial benefits in animal models of atherosclerosis, stroke, diabetes, and cerebral and cardiac I/R.^27^, ^28^, ^29^, ^30^ Here, we provide new evidence that HNG inhibits platelet activation and thrombus formation. The antiplatelet effect by HNG is present regardless of the type of agonist, suggesting that the potential target was probably not confined to any single receptor pathway. Subsequent functional analyses demonstrated that HNG promoted platelet microtubule stabilization. Consistently, HNG attenuated tubulin deacetylation and platelet shape change after activation. Although microtubules are well‐established drug targets in cancer, their roles in platelets remain far more elusive.^31^

Microtubule‐interfering agents showed consistent antimitotic functions in tumour cells, whereas their effects vary vastly in platelets.^32^, ^33^, ^34^ For instance, colchicine disturbs microtubule assembly and inhibits platelet release, although changes in granule secretion were not observed in vinblastine‐treated platelets.^35^ An early study showed that the colchicine effect could be reversed by stabilizing platelet microtubules with D2O, which alone may enhance platelet aggregation induced by calcium ionophore.^36^ Paclitaxel, another widely used microtubule‐stabilizing drug, shows a concentration‐dependent inhibition of platelet aggregation and secretion.^37^ These controversial results may be attributed to the differences in pharmaceutic mechanisms or may be explained by mechanisms beyond microtubule stabilization. Additionally, some studies argued that microtubules might not be required for granule secretion.^38^, ^39^, ^40^ However, TUBB1 knockout in vivo leads to spherical platelets, impaired aggregation and reduced granule secretion. Furthermore, a mutation impairing β1‐tubulin assembly was also shown to reduce platelet dense granule secretion, aggregation and collagen adhesion.^41^ Deletion of RanBP10, a β1‐tubulin‐binding protein, promotes microtubule stabilization and inhibits platelet aggregation and shape change, whereas adhesion and secretion were not affected. Our results showed that HNG not only inhibited platelet activation but also suppressed granule secretion. In line with our finding in platelets, HNG has been shown to suppress oxidative stress in cardiomyocytes^25^ and to inhibit ERK1/2 and AKT phosphorylation in neurons and the brain.^27^, ^42^ Phosphorylation of P38MAPK was not changed in either neurons or platelets treated with HNG. These data support the role of HNG in organ protection.

For the first time, we showed that HNG might stabilize platelet microtubules. Our findings may help to explain both the acute and chronic neuroprotection by HNG observed in myocardial I/R and AZD. First, HNG may ameliorate platelet hyperactivation induced by I/R, thereby functioning as a chaperone, which prevents the misfolding and thus formation of Aβ oligomers. The resulting lower circulating Aβ burden will reduce its deposition in the brain, which may subsequently attenuate microtubule depolymerization and tau hyperphosphorylation. The latter effect may be secondary to microtubule stabilization, as previous reports suggested that Taxol might also inhibit the tau hyperphosphorylation induced by Aβ.^43^ Thus far, whether HNG directly affects tau remains to be elucidated. On the other hand, chronic infusion of HNG is likely to benefit both platelets and neurons, thereby offering sustained protection against neurodegeneration via simultaneously blunting the circulating and topical pool of Aβ and other potentially detrimental releasates. Alternatively, the protective HNG may be ascribed to a marked decrease of the total Aβ levels, likely the consequence of the HNG‐induced overexpression of the Aβ‐degrading enzyme neprilysin. Neprilysin is an amyloid‐β peptide (Aβ)‐degrading enzyme, which declines in the brain during ageing, and then leads to a metabolic Aβ imbalance.^44^ However, the underlining mechanisms of HNG mediating neprilysin activity remain unclear.

Interestingly, microtubules have long been known as a key factor for AZD and are recently emerging as potential targets for thrombotic disease.^45^ Regardless, available candidates for enhancing microtubule assembly are mostly chemotherapeutic agents or nonphysiological synthetic peptides.^46^, ^47^, ^48^ Their application tends to be hampered by potential cytotoxicity and immunoneutralization. In addition, a higher incidence of cardiovascular events is noted in the AZD population, calling for an efficient prophylactic intervention.^49^ Unfortunately, clinical studies using aspirin have not presented any benefit in controlling AD.^50^ This could be due to aspirin‐insensitive pathways that contribute to AZD pathology. Mechanistically, inhibitors of integrin α_IIb_β_3_ may be effective but are not intended for prolonged use, as life‐threatening bleeding may occur. Double antiplatelet therapy using aspirin and P2Y_12_ inhibitors appears to be an alternative solution via blocking multiple platelet pathways involved in AZD, albeit increased bleeding risks and costs are limitations.^51^ Thus, our finding of HNG as a dual microtubule‐stabilizing and antiplatelet agent suggests that it may become a reasonable candidate for thrombotic comorbidities in patients with AZD. Alternatively, HNG may also yield instant and long‐lasting benefits in preserving organ function during and following cardiovascular I/R, probably through alleviating vascular inflammation and improving microcirculation. Future development of a more stable and affordable version of HNG will facilitate its translation into clinical application.

In summary, the results presented in this study demonstrated that HNG inhibits platelet activation and thrombus formation, potentially through stabilizing microtubules. Additionally, the therapeutic role of HNG in cardiovascular comorbidities of AZD requires further evaluation.

## CONFLICT OF INTEREST

The authors declare no conflicts of interest.

## AUTHOR CONTRIBUTION

LR, QL and XZ performed the experiments; LR, TY, LZ, XX and CT designed the experiments; LR, TY, QL and LZ analysed and interpreted the data; CT, TY, LR and LZ wrote the paper.

## Supporting information

Fig S1

Fig S2

Fig S3

## Data Availability

The authors confirm that the data supporting the findings of this study are available within the article and its supplementary materials.
