# Supplementary figures and images for: Humanin analogue, HNG, inhibits platelet activation and thrombus formation by stabilizing platelet microtubules
Source: J Cell Mol Med. 2020 Mar 16;24(8):4773–83. doi: 10.1111/jcmm.15151 (PMC7176859; doi:10.1111/jcmm.15151)

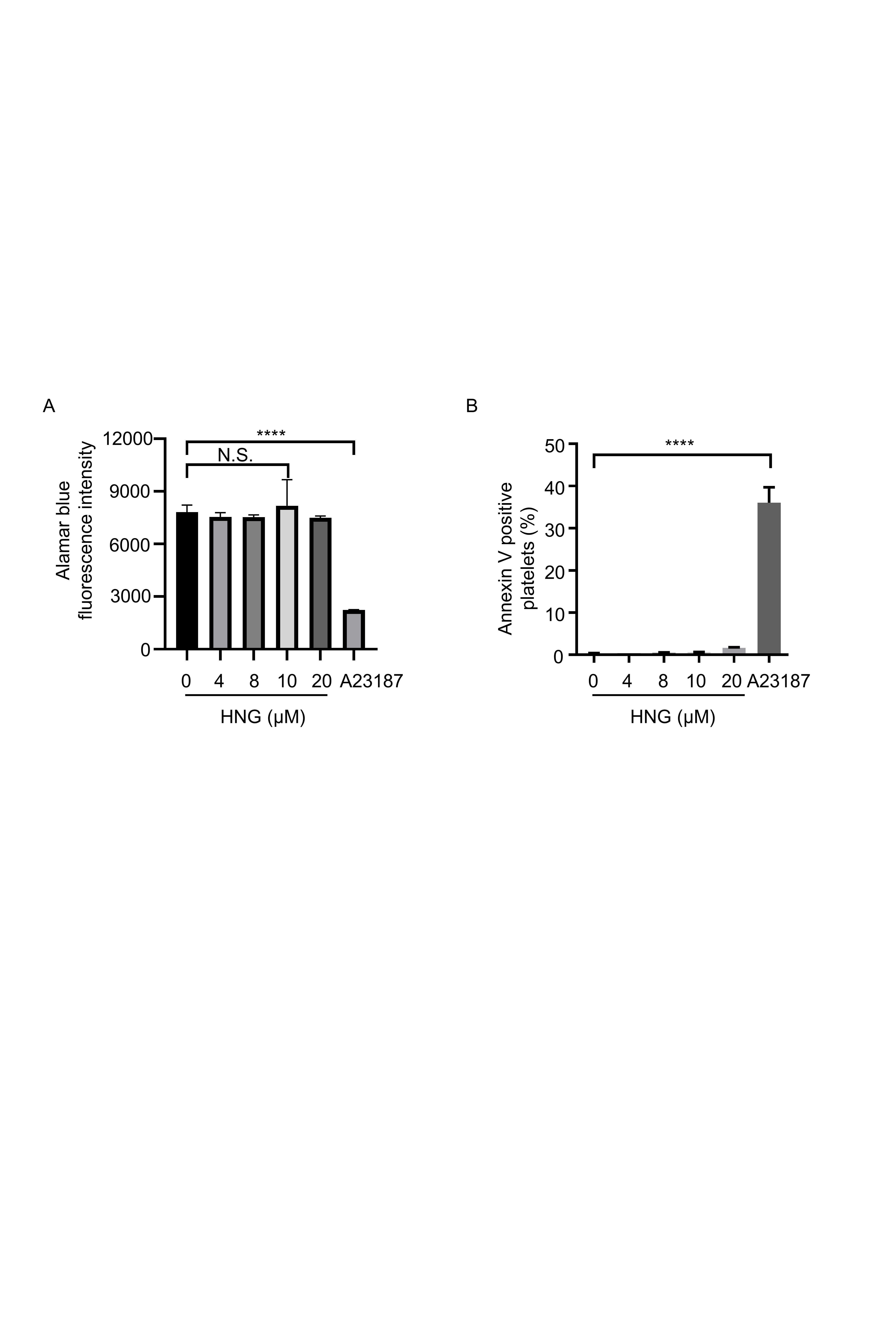

Supplement: Supplementary file 1 — Fig S1 [file JCMM-24-4773-s001.tif]

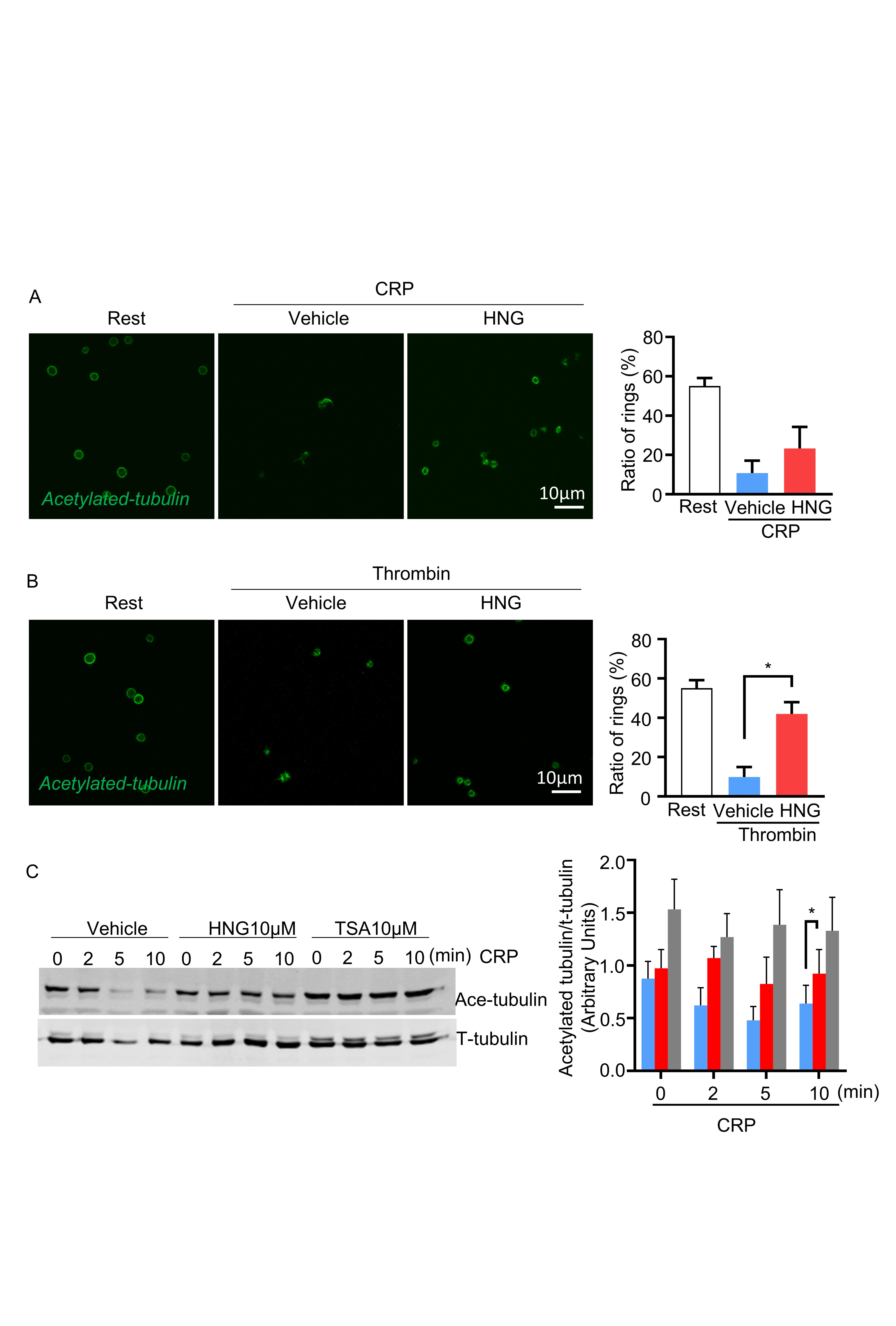

Supplement: Supplementary file 2 — Fig S2 [file JCMM-24-4773-s002.tif]

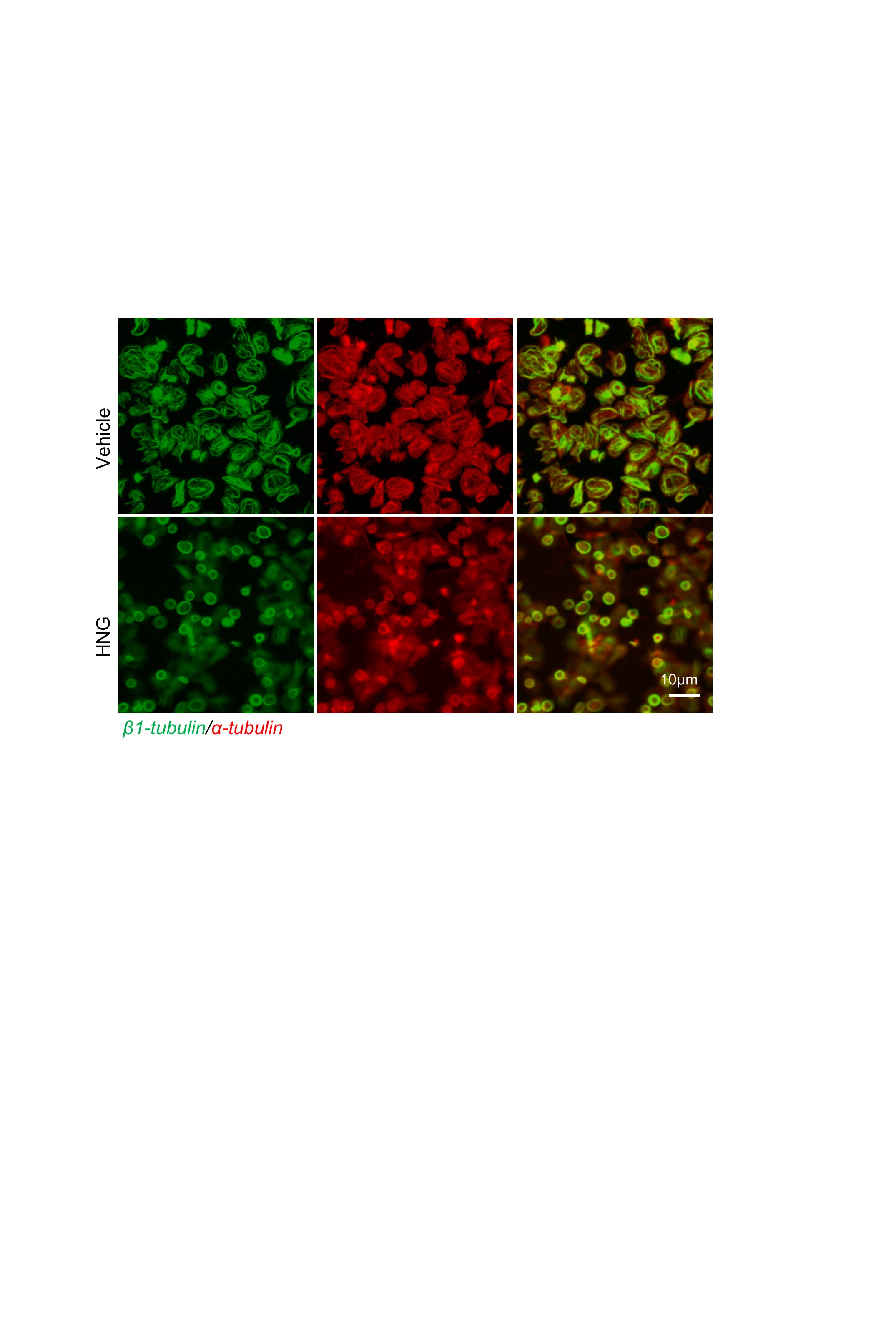

Supplement: Supplementary file 3 — Fig S3 [file JCMM-24-4773-s003.tif]
